# Golf Swing Segmentation from a Single IMU Using Machine Learning

**DOI:** 10.3390/s20164466

**Published:** 2020-08-10

**Authors:** Myeongsub Kim, Sukyung Park

**Affiliations:** Department of Mechanical Engineering, Korea Advanced Institute of Science and Technology (KAIST), Daejeon 34141, Korea; myeongsub@kaist.ac.kr

**Keywords:** golf, swing, sports, phase, segmentation, wearables, MEMS IMU, machine learning

## Abstract

Golf swing segmentation with inertial measurement units (IMUs) is an essential process for swing analysis using wearables. However, no attempt has been made to apply machine learning models to estimate and divide golf swing phases. In this study, we proposed and verified two methods using machine learning models to segment the full golf swing into five major phases, including before and after the swing, from every single IMU attached to a body part. Proposed bidirectional long short-term memory-based and convolutional neural network-based methods rely on characteristics that automatically learn time-series features, including sequential body motion during a golf swing. Nine professional and eleven skilled male golfers participated in the experiment to collect swing data for training and verifying the methods. We verified the proposed methods using leave-one-out cross-validation. The results revealed average segmentation errors of 5–92 ms from each IMU attached to the head, wrist, and waist, accurate compared to the heuristic method in this study. In addition, both proposed methods could segment all the swing phases using only the acceleration data, bringing advantage in terms of power consumption. This implies that swing-segmentation methods using machine learning could be applied to various motion-analysis environments by dividing motion phases with less restriction on IMU placement.

## 1. Introduction

To analyze the golf swing from a simple micro-electro-mechanical system (MEMS) inertial measurement unit (IMU)-based wearable device, it is necessary to classify and divide swing phases using IMU data. For convenient swing analysis in the field and driving range, MEMS IMU-based wearable devices on clubs, gloves, wrists, heads, and waists are widely used [1,2,3,4,5]. To analyze the golf swing using these devices, the time information of each swing phase must be first determined. The timing and length of the swing phases are used as indices during the swing analysis [6]. In addition, the golf swing is usually analyzed using the phases and their dividing points [7,8] because these phases have a certain pattern [9].

Various studies regarding golf swing analysis based on IMUs have primarily used simple and intuitive heuristic signal processing methods to divide swing phases, but the accuracy has not been sufficiently verified. Hsu et al. proposed a method of classifying the backswing (BS), downswing (DS), and follow-through (FT) phases using the magnitude threshold of the signal from a six-axis IMU attached to a golf club [10]. The errors of the phase length compared to the data from the optical motion capture system were reported to be 0.03 to 0.06 s, but the method was not verified with the number of subjects and swing trials. Nam et al. used thresholds of the signals from the IMU attached to the middle of the club shaft to determine the phase dividing points—address (ADD), backswing top (BST), impact (IMP), and finish (FIN)—but did not verify the accuracy of the calculation [3]. Lai et al. used the maximum negative peak of the data from IMUs attached to various parts of the body, including the left hand, left arm, pelvis, and upper body, to synchronize multiple sensors and compare subjects [4]. Jensen et al. estimated the BST timing of putting using zero-crossing timing of the *y*-axis angular velocity from a six-axis IMU attached to the putter but did not verify the accuracy [11]. Kooyman et al. also used the zero-crossing timing of the angular velocity perpendicular to the putter head from the IMU attached to the putter head to divide swing phases, but the accuracy was not verified [12].

Recently, due to the development of computing power, many studies have used machine learning approaches that can automatically learn the features of data from IMUs to increase the accuracy of general human activity recognition [13,14] and the classification and segmentation of sports motions [15], but no attempt has been made to apply models to estimate and divide golf swing phases. Several studies that have used IMUs to recognize general human motion have reported that using convolutional neural networks (CNNs) [16] is a superior approach to conventional machine learning algorithms [17,18,19,20,21]. Specifically, the CNN-based methods, which can automatically extract discriminative features with convolutional kernels, have enhanced or demonstrated better performances than conventional methods using predesigned features [17], mean and variance [18,20], support vector machine [18,19], 1-nearest neighbor [18], the 1-nearest neighbor with dynamic time warping [21], multilayer perceptron [19,21], restricted Boltzmann machine [20], and deep belief network [18]. In sports motion recognition studies using CNN, Anand et al. classified and detected the type of shots during tennis, badminton, and squash [22]. Jiao et al. classified the swing shape of the golf swing using multiple sensors [23]. Kautz et al. detected and classified player actions in beach volleyball [24], and Rassem et al. classified cross-country skiing movements [25]. Several studies using long short-term memory (LSTM) [26] or bidirectional LSTM (BLSTM) [27] recurrent neural networks (RNN) in sports motion recognition have exhibited similar or better performance compared to CNNs while classifying the type of swing motion [22] and cross-country skiing movement [25]. In golf swing motion, Jensen et al. estimated the lengths of the putt phases and various indicators using hidden Markov models from the six-axis IMU attached to the putter head; however, only the accuracy of the putt detection was reported to be 68.2%, and the accuracy of each swing-phase length was not reported [28]. Jiao et al. classified the swing shape with 95% accuracy using a CNN, two strain gauges, a three-axis accelerometer, and a three-axis gyroscope attached to the club [23]. Taken together, no study has segmented the swing phases of the golf swing using IMU with machine learning approaches, verifying the accuracy.

During the golf swing, constant time-series characteristics suggest the possibility of estimating the time information from an IMU attached to part of the body. Meister et al. reported that rotational biomechanical factors were highly consistent among professional golfers [29], which implies that some constant time-series characteristics exist during a golf swing. Many studies that have analyzed the golf swing motion of the multi-segmented human body using experiments or simulations have reported that the body segments generate the golf swing motion with a proximal to distal sequence [30,31,32,33,34,35]. Verikas et al. reported stable individual sequences in activation profiles of the arm and shoulder muscles [36]. Their results revealed that the “effective profiles started with a common avalanche effect from a triad of muscles (right *rhomboideus*, right *trapezius*, left *rhomboideus*) followed by the simultaneous activation of another triad of muscles (right *flexor carpi radialis*, left *extensor digitorum communis*, and left *trapezius*)” [36]. Based on these existing studies, we can estimate the time information of a golf swing from the motion information of any part of the body by learning prior knowledge of certain time-series features, including the sequence of body segments.

Therefore, in this study, we proposed new methods for the golf swing segmentation from a single IMU attached to each body segment. This was performed using the BLSTM and CNN machine learning models that can automatically learn the characteristics of the time-series data, including the sequence of the body segments.

## 2. Methods

In this study, we proposed a BLSTM-based and a CNN-based method for estimating the time points to divide the golf swing phases from each IMU attached to the left wrist, the right side of the head, and the right side of the waist. We conducted a golf swing experiment to train and verify the methods, comparing the estimation results with the existing heuristic approach. First, we define the golf swing phases used in this study and describe the details of the golf swing experiment and data processing procedures. Then, we explain the proposed BLSTM-based, CNN-based estimation methods, and heuristic estimation method for comparison. Finally, we describe how to train and verify the proposed methods in this study.

### 2.1. Golf Swing Phases

**Figure 1:**
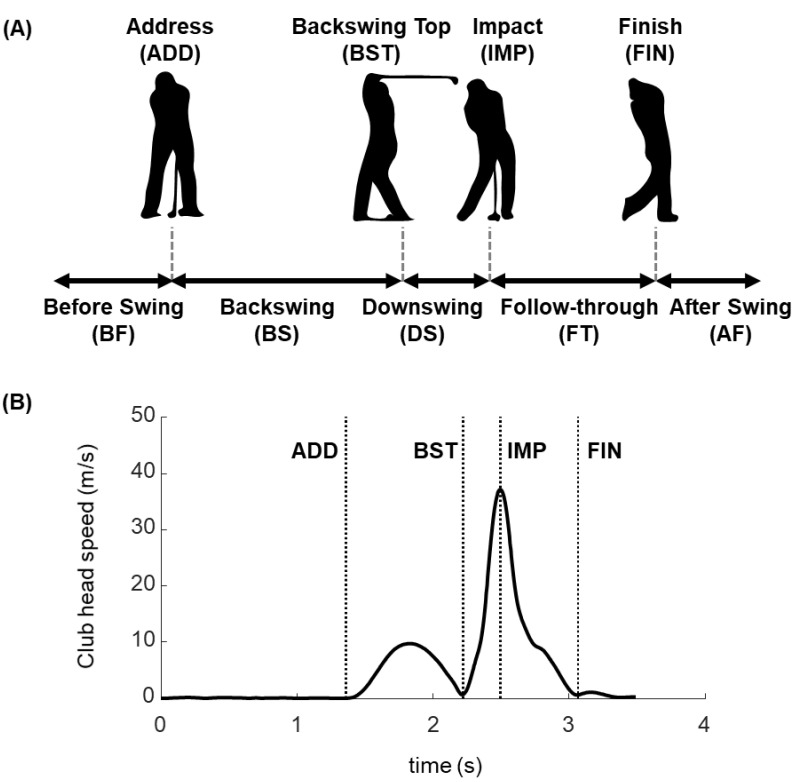
(**A**) A schematic of a full golf swing illustrating the phases and dividing points. (**B**) Clubhead speed with phase dividing points: five phases, including before swing (BF), backswing (BS), downswing (DS), follow-through (FT), and after swing (AF), with four dividing points of address (ADD), backswing top (BST), impact (IMP), and finish (FIN). Dividing points are defined with the clubhead position and clubhead speed represented in (B).

Golf swing phases can be divided using several criteria based on the club [9,32] or body [37,38] motions. In this study, we divided a full golf swing with clubhead motion into five phases: before the swing (BF), BS, DS, FT, and after the swing (AF), with four dividing points: ADD, BST, IMP, and FIN (Figure 1A). In addition, ADD is when the clubhead speed is the local minimum just before the BS. The BST is when the clubhead speed is the local minimum right after the BS. Moreover, the IMP is when the clubhead passes the marker on the ground aligned with the golf ball during the DS. Finally, FIN is when the clubhead speed reaches the local minimum again after the FT (Figure 1B).

### 2.2. Experiment

**Figure 2:**
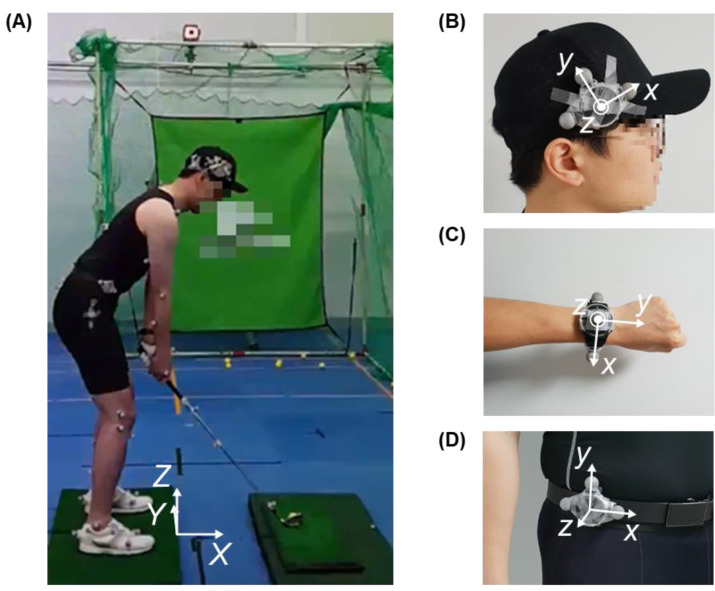
Wearable devices (T5, VC Inc., South Korea) with micro-electro-mechanical system (MEMS) inertial measurement unit (IMU) attached to each body part: (**A**) full body of the subject at address, (**B**) head attachment, (**C**) left wrist attachment, and (**D**) waist attachment. The global coordinate was set based on the subject, as shown in (**A**); the *X*-axis is the forward direction of the subject, the *Y*-axis is in the direction of the left side of the subject, and the *Z*-axis is the vertically upward direction. The local coordinates of the wearable devices were set, as shown in (**B**–**D**).

Nine male professional golfers belonging to the Korea Professional Golfers′ Association or Korea Golf Federation and eleven male amateur golfers with an average handicap (additional required strokes compared to par in 18 holes; professional golfers do not track their handicap) of 15.3 ± 4.3 participated in the experiment under the approval of the KAIST Institutional Review Board (KH2018-85 and KH2019-140). Twenty subjects were right-handed, with an average age of 41.2 ± 9.1, an average height of 175.4 ± 6.9 cm, and an average weight of 79.9 ± 13.4 kg. Each subject swung 10 times using a driver club and 10 times using a 7 iron club, swinging once after a light jump in place to align the time axis of the sensor data, hitting a training sponge golf ball in a laboratory environment. We collected three-axis acceleration and three-axis angular velocity from each wearable device (T5, VC Inc., Seoul, Korea) attached to the left wrist, head, and pelvis of the subject (Figure 2). The MEMS IMU (BMA456, BMG250, Bosch Sensortec GmbH, Reutlingen, Germany) was built into the wearable device and set to a range of ±16 g and 2000 dps, respectively, and the data were collected at a sample rate of 200 Hz. The range of the IMU was determined with the peak acceleration and the peak angular velocity during the golf swing, and the sample rate was determined considering the general MEMS IMU-based wearable devices [15]. For the reference of the swing motion, we used an optical motion capture system. A total of 62 optical markers were attached to the subject, club, IMUs, and ground to collect the reference motion data. Optical markers attached at the end of the head-side of the club shaft and on the ground about 10 cm away on the global *X*-axis from the golf ball were used to calculate the reference swing phases. Fourteen motion capture cameras (Hawk and Eagle, Motion Analysis, Rohnert Park, CA, USA) collected the motion data during each swing at a sample rate of 200 Hz.

### 2.3. Data Processing

Out of the collected data on the 400 swings from the experiments, 389 swing data points were used, excluding 11 swings that had measurement failure. The collected data were transformed into an input dataset and were labeled for model training and verification through the following data processing procedure.

The data collected from the IMUs and the motion capture cameras filtered using a bidirectional Butterworth 10th low-pass filter with a cutoff frequency of 10 Hz, determined from a power spectral density analysis using Fourier transform [39]. For the time alignment between the data from the IMUs and the motion capture cameras, we used the peak of acceleration during the jump motion before each swing. Each swing-phase dividing point: ADD, BST, IMP, and FIN, was calculated from the reference motion data of the clubhead collected from the motion capture camera. To apply the proposed method, the data collected from wearable devices must first be cut and imported. Considering the peak of the acceleration signal, which is relatively clearly seen at the impact during the full golf swing motion, we cut each swing to a total of 3.5 s from 2.5 s before to 1 s after the IMP point.

**Figure 3:**
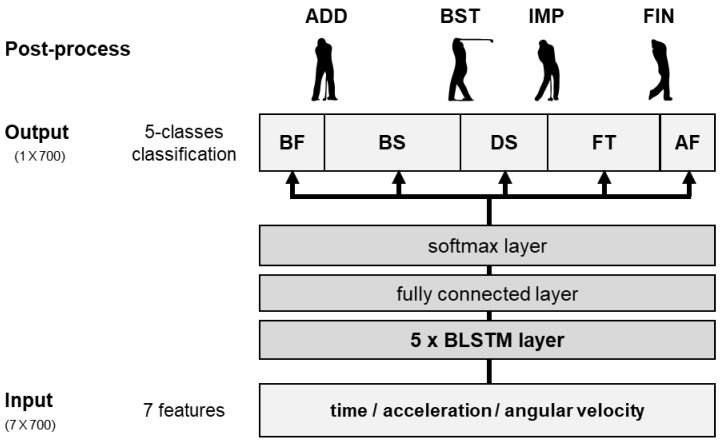
The architecture of the proposed bidirectional long short-term memory (BLSTM)-based golf swing segmentation method. Five phases were before swing (BF), backswing (BS), downswing (DS), follow-through (FT), and after swing (AF). Four phase dividing points were the address (ADD), backswing top (BST), impact (IMP), and finish (FIN).

**Figure 4:**
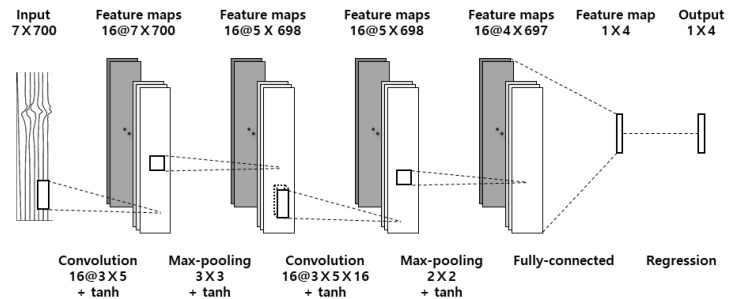
The architecture of the proposed convolutional neural network (CNN)-based golf swing segmentation method.

For the BLSTM-based model, a 7 × 700 matrix with seven features of time, local three-axis acceleration, and local three-axis angular velocity was set as the input (Figure 3). A 1 × 700 matrix consisting of five classes of swing phases: BF, BS, DS, FT, and AF, at each time sample, was set as the label. Input data were normalized so that each feature fell within the range of −1 to +1. For the CNN-based model, the same input matrix, as for the BLSTM-based model, was used (Figure 4). A 1 × 4 matrix with four swing-phase dividing points: ADD, BST, IMP, and FIN, was set as the label. Input data were also normalized within the range of −1 to +1. When using only the acceleration or only the angular velocity data, both models set the input as a 4 × 700 matrix, with four features of time and three-axis either acceleration or angular velocity.

### 2.4. Swing-Phase Segmentation Algorithms

#### 2.4.1. Heuristic-Based Segmentation

To compare the accuracy of the proposing swing-phase segmentation algorithms, we made a handcrafted heuristic-based segmentation algorithm using the minimum, maximum, and zero-crossing points of three-axis acceleration and three-axis angular velocity from IMUs, similar to previous studies [3,4,10,11,12]. Thirty indicator candidates were selected from the IMU data of the wrist, head, and pelvis to estimate each swing-phase dividing point. Out of 30 indicator candidates, 12 indicators with the smallest estimation error were selected as segmentation indicators for the heuristic-based algorithm (Table 1).

#### 2.4.2. Bidirectional Long Short-Term Memory-Based Method

The proposed BLSTM-based swing-phase segmentation algorithm consisted of an input layer, five BLSTM layers with 32 hidden units for each, a fully connected layer, and a softmax output layer [40]. The output layer revealed one of five swing-phase classes for each time sample. The algorithm finally converted the phases into swing-phase dividing points, where the classes changed from one to another (Figure 3). Model parameters were determined with trial and error.

#### 2.4.3. Convolutional Neural Network-Based Method

The proposed CNN-based swing-phase segmentation algorithm consisted of an input layer, two CNN layers (convolution layer, tanh layer, max-pooling layer, and tanh layer again), a fully connected layer, and a regression output layer. The output layer revealed four swing-phase dividing points directly (Figure 4). Model parameters were determined with trial and error.

### 2.5. Model Training and Validation

To validate the swing segmentation accuracy of the proposed methods, we compared the phase dividing points from the reference. The proposed methods were verified using leave-one-out cross-validation. To test the swing data of one subject, each proposed method was trained using the swing data of the remaining 19 subjects, and the test was repeated for all 20 subjects to obtain a total average of errors and the between-subject variance. To train the models, 90% of the 19 subjects’ swing data was randomly divided into a training set, and the remaining 10% was divided into a validation set. The training was terminated using validation stopping, and the adaptive moment estimation (Adam) optimizer [41] was used. We used MATLAB R2019a software and the Deep Learning Toolbox (MathWorks Inc., Natick, MA, USA) to establish, train, and test the proposed models.

## 3. Results

**Figure 5:**
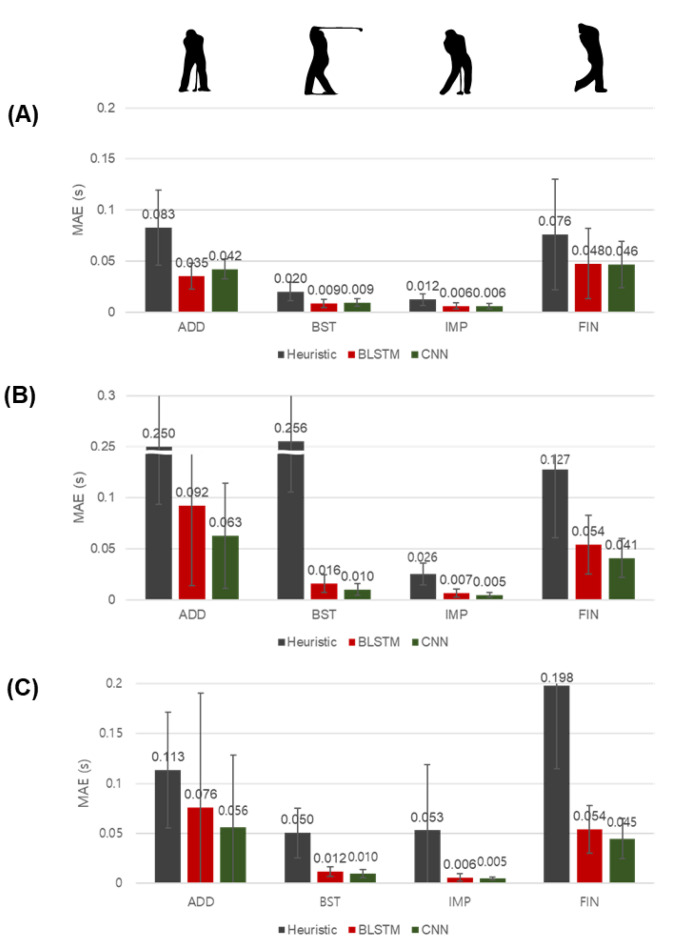
Mean absolute error (MAE) of phase dividing point estimations from each IMU attached to the (**A**) wrist, (**B**) head, and (**C**) waist. Phase dividing points were the address (ADD), backswing top (BST), impact (IMP), and finish (FIN). The heuristic (gray) and proposed bidirectional long short-term memory (BLSTM)-based (red) and convolutional neural network (CNN)-based (green) methods were compared. The error lines represent the standard deviation for each subject.

The proposed BLSTM-based and CNN-based methods estimated all four major swing-phase dividing points from the IMU attached to the wrist with a lower mean absolute error (MAE) than the heuristic method (Figure 5A). The proposed methods also effectively estimated all dividing points from each IMU attached to the head and waist with a low MAE compared to the heuristic method (Figure 5B,C).

**Figure 6:**
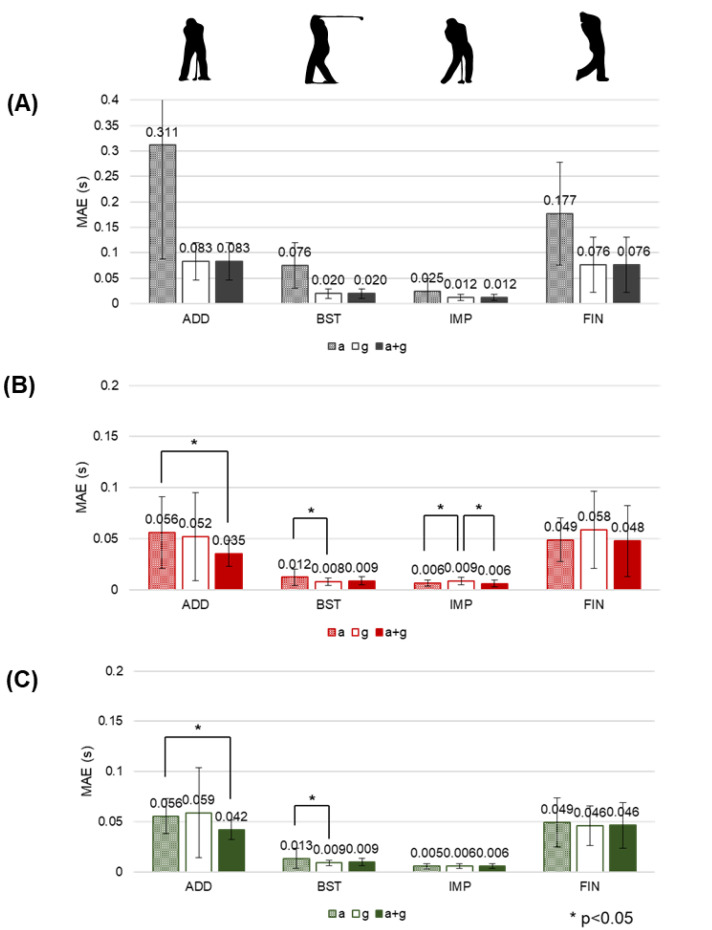
Mean absolute error (MAE) of phase dividing point estimations from a single IMU attached to the wrist with (**A**) heuristic, (**B**) bidirectional long short-term memory (BLSTM)-based, and (**C**) convolutional neural network (CNN)-based methods. Phase dividing points were the address (ADD), backswing top (BST), impact (IMP), and finish (FIN). Results of using only acceleration (“a” hatched), only angular velocity (“g” blank), and both acceleration and angular velocity (“a+g” solid) were compared. The error lines represent the standard deviation for each subject. Asterisk shows statistical significance (*p* < 0.05).

The proposed methods could lower the estimation error using both acceleration and angular velocity data and could accurately estimate using only the acceleration data from the IMU attached to the wrist (Figure 6). In the case of the heuristic method, we only used either the acceleration or angular velocity data separately, and the MAE of the estimation from the angular velocity was lower than the estimation from the acceleration in all four phase dividing points (Figure 6A). With the proposed BLSTM-based and CNN-based methods, using both acceleration and angular velocity together showed a significantly lower MAE at several phase dividing points compared to using each of them to estimate (Figure 6B,C). Phase dividing points estimation using only the acceleration data with the proposed methods showed a lower MAE than the estimation using both the acceleration and angular velocity data with the heuristic method (Figure 6).

The proposed methods revealed more accurate phase length estimations compared to the heuristic method. The average length of each swing phase calculated from the reference is shown in Table 2. We compared each swing phase length calculated from the phase dividing points estimated using the heuristic method and the proposed methods (Table 3). The errors of all swing phases were about 2.7% to 9.0% on average using both the acceleration and the angular velocity data, reducing about half of the segmentation errors compared to the heuristic method. In the case of the wrist IMU, the errors using only the acceleration data to segment the DS phase were about 4.8% on average with the BLSTM-based method, and about 3.7% on average with CNN-based method, respectively, which were accurate compared to the heuristic method. In the case of the head and waist IMUs, the errors of all swing phases using only acceleration with the proposed methods were about 8.3% to 13.2% on average, accurate compared to the heuristic method using both the acceleration and the angular velocity. One exception was the BS from the waist IMU, for which the BLSTM-based method showed a similar error with the heuristic method.

## 4. Discussion

In this study, we proposed and verified a machine learning-based method for golf swing segmentation using a single IMU attached to each body segment. The results, generated using the IMU data collected from the wrist, head, and waist, showed accurate swing segmentation compared to the heuristic-based method. We employed both a CNN-based method and a BLSTM-based method and suggested the CNN-based method for future studies. In our work, the DS was the most accurately estimated phase. Importantly, the proposed methods are capable of segmenting a golf swing using only acceleration data.

Our proposed methods are applicable even with each IMU attached to other parts of the body rather than the club or the wrist. The clubhead motion divides the golf swing phases, and because the hand and the wrist are closest to the clubhead, they move similarly. Thus, the heuristic method provided much more accurate golf swing segmentation at the wrist (Figure 5A). However, it was difficult to employ the heuristic method as the time delay of the motion between the clubhead and the body parts increased. Consequently, golf swing segmentation via the heuristic method became more erroneous using IMU data from the head and waist rather than the wrist (Figure 5B,C). On the other hand, the proposed BLSTM-based and CNN-based methods demonstrated accurate segmentation compared to the heuristic method because they were able to learn the specific features of the sequential motion (Figure 5B,C). While the MAE of the heuristic method using the head and waist IMU was approximately double the MAE associated with using the wrist IMU, the proposed BLSTM and CNN methods resulted in similar errors using each of the wrist, head, and waist IMU, except for the ADD (Figure 5). In the case of ADD estimation, subject 20 was an outlier with an error of 0.389 s (head) and 0.571 s (waist) on average with the BLSTM-based method and 0.277 s (head) and 0.368 s (waist) on average with the CNN-based method. It seems that the sequential characteristics of the whole-body motion of subject 20 during the ADD was significantly different from the other subjects. The phase length errors of the BLSTM-based and CNN-based methods using each IMU attached to the head and waist also demonstrated accurate estimation with errors of 4.6% to 9.0% on average for all phases, while the heuristic method resulted in errors of 11.5% to 79.1% on average (Table 3). Therefore, unlike previous studies that distinguished swing phases from an IMU attached directly to a club [3,10,11,12,28,42] or from image or video data [43,44], our methods are capable of segmenting swing phases using only a single IMU sensor attached to each body segment: head, wrist, and waist.

The proposed methods can accurately divide the DS phase, which is considered the most interesting and important phase of the golf swing [9,45,46], estimating the phase dividing points with an MAE up to 16 ms on average and the phase length error up to 5.2% on average using IMU data collected at the wrist, head, and waist. Because golf is a sport in which players compete with each other with the number of total strokes, the clubhead speed (determining the distance of the ball flight) coming from the DS phase is a key element of a good golf swing. Therefore, the DS phase is the most important phase to be accurately segmented prior to further analysis. Currently, the sample rates of general MEMS IMU-based wearable devices are 10 to 500 Hz [15]. In this study, we collected all the data with a sample rate of 200 Hz; importantly, the methods used can estimate the BST and IMP with errors of 5 to 16 ms on average, or about 1 to 3 sample steps. However, in the case of the ADD and FIN, the errors were 41 to 92 ms on average, much higher than the BST and IMP. The ADD and FIN were difficult to estimate using both our proposed methods and the heuristic method because the movement and the following kinematic signals appeared small relative to the noise at the starting and finishing points of the golf swing. Consistent with our findings, Mesaros et al. also demonstrated that acoustic event classification performance decreased considerably with a lower signal-to-noise ratio [47]. Our methods estimated all the dividing points with each single trained model, which may miss small features at the ADD and FIN. Thus, to improve the estimation accuracy at the ADD and FIN, we may focus on these specific dividing points when constructing the structure of the estimation model. The BLSTM-based model could be modified as classifying the motion within only two classes, before and after the ADD, rather than classifying the motion within five different phases. The CNN-based model may set the convolution layers to learn only the features of the ADD, regressing only the ADD point as the output label. These modifications will help the model to concentrate on the small features in the kinematic data with low signal-to-noise ratios.

The CNN-based method is more suitable for golf swing segmentation than the BLSTM-based method. In sequence modeling, various studies have used convolutional networks [48,49,50,51,52] and recurrent networks [53,54,55]. In the case of the time series motion data in this study, the motion was determined under the mechanical system of the human body in the time axis, and the feature information also appeared in the two-dimensional matrix form. Therefore, both CNN-based models, which extract and learn the features, and RNN(or especially BLSTM in this study)-based models, which learn temporal information in the sequence, have been used in previous studies [17,18,19,20,22,23,25]. Anand et al. reported similar accuracies of the CNN-based and BLSTM-based methods for shot classification [22], and Rassem et al. reported that the LSTM-based method was better than the CNN-based method for classifying cross-country skiing gears [25]. However, in contrast with the studies of Anand et al. and Rassem et al., our results showed that the CNN-based method slightly outperformed the BLSTM-based method for the golf swing segmentation at the head and waist (Figure 5B,C). In a study by Bai et al., who empirically compared convolutional and recurrent networks in a wide range of tasks and datasets, simple convolutional architecture generally outperformed LSTM, leading the authors to conclude that convolutional networks should be considered as a natural starting point for sequence modeling tasks [56], which is consistent with our results. Further, Bai et al. showed that the convolutional architecture maintained accuracy regardless of the sequence length, whereas the LSTM model quickly degenerated to random guessing as the sequence length grew [56]. Thus, the performance of the LSTM-based method may decline as the sequence length increases, as the sample rate increases, or the target movement becomes longer in time series motion data. Our proposed BLSTM-based method classified 700 time samples with five phase classes, while the CNN-based method estimated four-phase dividing points directly. Therefore, the BLSTM-based method needs to estimate more outputs and requires an additional post-process, which was not considered during the model training procedure, to calculate the phase dividing points. These structural differences may have led to differences in accuracy between the BLSTM-based and the CNN-based methods. Finally, we compared the computational efficiency of the proposed methods. The number of parameters of both the BLSTM-based and the CNN-based methods is approximately 100 K. However, under the same computing power of a 3.5 GHz Intel Core i5-7600 central processing unit, the BLSTM-based method takes approximately 200 s, while the CNN-based method needs approximately 30 s. The computation times of the proposed methods are about 0.179 s and 0.040 s, respectively, about 60 times and 13 times compared to the heuristic method that takes about 0.003 s. Both proposed methods seem applicable to the golf wearables considering that the current wearable devices offer over 1.0 GHz central processing unit [57], and we need the computation only once for each golf swing. Still, the CNN-based method is advantageous in terms of power consumption due to the computing efficiency compared to the BLSMT-based method. Taken together, considering both the accuracy and the computing efficiency, we recommend using the CNN-based approach for swing segmentation using an IMU attached to the head, waist, or wrist.

The proposed methods are capable of segmenting the golf swing using only acceleration data and can improve the accuracy by using both acceleration and angular velocity data together. The acceleration signal alone can be used in our proposed methods to estimate the phase segmentation with higher accuracy than a heuristic method, resulting in reduced power consumption, which is important for MEMS IMU-based wearable devices [1,58,59]. The results in Figure 6B,C showed an MAE of 5–60 ms using only acceleration data, which were more accurate than the heuristic method that resulted in an MAE of 20–300 ms with acceleration data alone and an MAE of 10–80 ms with both acceleration and angular velocity, as shown in Figure 6A. Comparing the phase length estimating results in Table 3, our methods resulted in phase errors of 8–13% on average using only acceleration data, indicating a significant improvement on the heuristic method that resulted in errors of 11–80% on average using both the acceleration and angular velocity. Further, the heuristic method can determine the phase dividing points depending on only one of the acceleration or angular velocity, while our proposed methods can consider both signals at the same time due to the automatic feature learning characteristics of the machine learning models. Thus, we further increased the estimation accuracy by considering two different physical quantities together. The proposed methods, using both the acceleration and angular velocity, significantly reduced the MAE at the ADD (Figure 6B,C). Rotational biomechanics have been identified as a key element influencing power generation during the golf swing [29]; thus, unsurprisingly, the heuristic method performed poorly at the segmentation task using only acceleration data. These results demonstrated that the CNN-based and BLSTM-based methods have the advantages of extracting and learning “hidden” features, which are difficult to extract directly with the heuristic approach and considering multiple signals at once.

Further verification of our results is required because this study was conducted under limited conditions and with a limited subject pool. To our best knowledge, this is the first attempt to compare the segmentation accuracy of golf swing phases from IMU data in detail. The heuristic algorithm for comparison in this study was limited to three basic types of indicators: minimum, maximum, and zero-crossing points. Golf swing data used in this study were collected with practice sponge balls in an indoor laboratory. Although no reports have been made of differences in body motion when hitting a practice sponge ball rather than a real golf ball, it is known that the clubhead speed decreases by about 30% when impacting a real golf ball [60]. The acceleration applied to the clubhead during IMP is transmitted to the body through the shaft. Thereafter, differences in the swing motion of the body segments during the FT phase may occur. The subjects who participated in the experiment were skilled right-handed male golfers with a handicap of 0 to 23. A wide range of subjects, including low-skilled, female, young, or senior golfers, were not considered in this study. Various studies have reported golf swing differences according to skill level [4,9,61]. In the case of female golfers, Zheng et al. reported differences in swing mechanics between Ladies Professional Golf Association Tour (LPGA) and Professional Golf Association Tour (PGA) golfers, especially at the wrist and elbow [62]. Parker et al. reported strong evidence that clubhead speed, time to arm peak speed downswing, and angular wrist peak speed were different between elite male and female golfers [63], and Horan et al. reported that skilled male and female golfers exhibited different kinematics in their thoracic and pelvic motions [64]. Younger golfers with a mean age of 21.1 years exhibit significant swing differences, reaching their peak downswing force later in the shot compared to senior golfers with a mean age of 65.7 years [65]. Therefore, to apply the golf swing segmentation methods proposed in this study to a general population, these methods should be verified using a wider range of subjects.

The swing-segmentation methods proposed in this study can be applied according to various motion analysis environments. Our methods can segment basic phase information from time-series IMU data and subsequently help to analyze the kinetics and kinematics of the motions. The methods could be applied to various general motions, including swinging, kicking, throwing, walking, and running motions, which contain certain time-series features and body-segment sequences. In addition, because the attachment location of the IMU to collect motion data for the proposed methods is not restricted, we can consider and expand IMU placements to the various body parts, depending on each application.

## 5. Conclusions

In conclusion, the proposed CNN-based and BLSTM-based machine learning methods can estimate and divide golf swing phases using kinematic data collected from a single IMU by learning time-series features, including the body sequence during the swing motion. These methods can segment the golf swing from IMUs attached to various body parts, eliminating the limitation of the sensor location for wearable applications. In addition, our methods can segment golf swing phases using only acceleration data, bringing advantage in terms of power consumption. These methods could be applied with less restriction on IMU placement to many human motion segmentation problems with body sequences.

## Figures and Tables

**Table 1:** Heuristic-based golf swing segmentation indicators.

IMU Placement.	ADD ^1^	BST ^1^	IMP ^1^	FIN ^1^
Wrist	*min.*^2^ of ω ^3^ in *y*-axis	*z.c.*^2^ of ω in *z*-axis	*min.* of ω in *y*-axis	*min.* of ω in *norm*
Head	*min.* of ω in *norm*	*min.* of *a* ^3^ in *x*-axis	*min.* of *a* in *norm*	*min.* of ω in *norm*
Waist	*min.* of ω in *y*-axis	*z.c.* of ω in *y*-axis	*z.c.* of *a* in *x*-axis	*min.* of ω in *norm*

^1^ ADD: address, BST: backswing top, IMP: impact, FIN: finish; ^2^ min.: minimum, z.c.: zero-crossing; ^3^ ω: angular velocity, a: acceleration.

**Table 2:** Phase lengths calculated from the reference data.

Backswing	Downswing	Follow-through	Full-Swing
1.163 ± 0.232 s	0.317 ± 0.050 s	0.670 ± 0.119 s	2.151 ± 0.294 s

**Table 3:** Phase length estimation errors (%) from the inertial measurement unit (IMU) attached to each body part for each phase.

Error (%)		Backswing			Downswing			Follow-through		
		a ^1^	g ^1^	a + g ^1^	a	g	a + g	a	g	a + g
	**H** ^3^	28.5 ± 7.5	6.7 ± 4.4	6.7 ± 4.4	24.3 ± 11.9	6.9 ± 2.3	6.9 ± 2.3	35.8 ± 17.5	10.9 ± 7.8	10.9 ± 7.8
**Wr** ^2^	**B** ^3^	4.9 ± 3.0	3.6 ± 2.8	3.1 ± 2.6	4.8 ± 2.7	3.2 ± 2.0	2.9 ± 1.9	9.1 ± 6.5	6.7 ± 6.0	7.1 ± 6.6
	**C** ^3^	4.9 ± 3.6	4.9 ± 3.3	4.5 ± 2.9	3.7 ± 2.1	3.2 ± 2.4	2.7 ± 1.8	6.8 ± 5.7	7.2 ± 6.0	6.9 ± 5.5
	**H**	N/A	N/A	34.3 ± 20.7	N/A	N/A	79.1 ± 33.4	N/A	N/A	20.5 ± 15.5
**Hd** ^2^	**B**	12.2 ± 5.7	8.9 ± 6.3	6.6 ± 4.3	8.4 ± 4.7	4.6 ± 3.6	4.6 ± 2.9	10.6 ± 8.0	8.7 ± 8.4	8.1 ± 7.8
	**C**	7.1 ± 5.4	5.9 ± 4.0	5.8 ± 3.9	5.8 ± 4.2	5.0 ± 3.5	3.3 ± 2.3	7.8 ± 6.0	7.4 ± 6.1	6.3 ± 5.5
	**H**	N/A	N/A	11.5 ± 5.9	N/A	N/A	26.3 ± 20.9	N/A	N/A	33.4 ± 18.2
**Wa** ^2^	**B**	12.5 ± 5.6	6.4 ± 3.7	6.7 ± 3.8	8.3 ± 4.9	4.6 ± 3.0	5.2 ± 3.6	13.2 ± 9.1	8.1 ± 6.7	9.0 ± 6.6
	**C**	6.6 ± 3.7	5.1 ± 3.3	4.2 ± 2.6	4.0 ± 3.0	3.4 ± 2.7	2.7 ± 2.1	8.5 ± 6.6	7.0 ± 5.8	6.4 ± 5.6

^1^ a: using only acceleration, g: using only angular velocity, a + g: using both acceleration and angular velocity; ^2^ Wr: wrist, Hd: head, Wa: waist; ^3^ H: heuristic, B: bidirectional long short-term memory, C: convolutional neural network.
